# Annual Atmospheric Corrosion of Carbon Steel Worldwide. An Integration of ISOCORRAG, ICP/UNECE and MICAT Databases

**DOI:** 10.3390/ma10060601

**Published:** 2017-05-31

**Authors:** Belén Chico, Daniel de la Fuente, Iván Díaz, Joaquín Simancas, Manuel Morcillo

**Affiliations:** National Centre for Metallurgical Research (CENIM/CSIC), Av. de Gregorio del Amo, 8, 28040 Madrid, Spain; bchico@cenim.csic.es (B.C.); delafuente@cenim.csic.es (D.d.l.F.); ivan.diaz@cenim.csic.es (I.D.); jsimancas@cenim.csic.es (J.S.)

**Keywords:** atmospheric corrosion, carbon steel, damage function, ISOCORRAG, ICP/UNECE, MICAT

## Abstract

In the 1980s, three ambitious international programmes on atmospheric corrosion (ISOCORRAG, ICP/UNECE and MICAT), involving the participation of a total of 38 countries on four continents, Europe, America, Asia and Oceania, were launched. Though each programme has its own particular characteristics, the similarity of the basic methodologies used makes it possible to integrate the databases obtained in each case. This paper addresses such an integration with the aim of establishing simple universal damage functions (DF) between first year carbon steel corrosion in the different atmospheres and available environmental variables, both meteorological (temperature (T), relative humidity (RH), precipitation (P), and time of wetness (TOW)) and pollution (SO_2_ and NaCl). In the statistical processing of the data, it has been chosen to differentiate between marine atmospheres and those in which the chloride deposition rate is insignificant (<3 mg/m^2^.d). In the DF established for non-marine atmospheres a great influence of the SO_2_ content in the atmosphere was seen, as well as lesser effects by the meteorological parameters of RH and T. Both NaCl and SO_2_ pollutants, in that order, are seen to be the most influential variables in marine atmospheres, along with a smaller impact of TOW.

## 1. Introduction

The economic impact of corrosion of metallic structures is a matter of great relevance throughout the world. The World Corrosion Organisation (WCO) currently estimates the direct cost of corrosion worldwide at between €1.3 and 1.4 trillion, which is equivalent to 3.8% of the global Gross Domestic Product (GDP). More than half of the considerable damage due to corrosion is a result of atmospheric impacts on materials, which is logical considering that most metallic equipment and structures operate in the atmospheric environment. For this reason, the action of the atmosphere on metals is one of the major issues in corrosion science.

In a perfectly dry atmosphere, metallic corrosion progresses at an extremely low rate, and for practical purposes can be ignored. However, on wet surfaces, corrosion can be quite severe, as the atmospheric corrosion process is the sum of the individual corrosion processes that take place whenever an electrolyte layer forms on the metal surface. However, for the corrosion rate to be really significant, the atmosphere must also be polluted. Of all atmospheric pollutants, chlorides from marine aerosol and sulphur dioxide (SO_2_) mainly from the combustion of fossil fuels, are the most common aggressive agents in the atmosphere.

It is a well-known fact, which has been proven by practical experience with real structure behaviour and the results of numerous tests, that the corrosion rate of metals in the atmosphere can be tens or even hundreds of times higher in some places than in others. Thus, it is of great interest to understand the basic variables that operate in atmospheric corrosion and in order to establish a classification of the aggressiveness of an atmosphere. The best possible knowledge of the factors that affect atmospheric corrosivity would obviously help to plan anticorrosive measures for metals in a given environment.

In the 1980s, three different cooperative studies involving the participation of a large number of countries were carried out:

- **ISOCORRAG cooperative programme.** This programme was designed by the Working Group/WG 4 of ISO 156 Technical Committee “Corrosion of metals and alloys”, with the aim of standardising atmospheric corrosion tests) [1]. The Programme began in the year 1986 and, as a result of the efforts of WG 4, four international standards were developed: ISO 9223 [2,3], ISO 9224 [4], ISO 9225 [5] and ISO 9226 [6]. These standards were based on an extensive review of atmospheric exposure programmes carried out in Europe, North America, and Asia. The aim of drawing up these documents was to establish simple and practical guidelines for the technicians responsible for designing structures to be exposed to the atmosphere and for corrosion engineers responsible for adopting anticorrosive protection measures. ISO 9223 [2] provided a general classification system for atmospheres based either on 1-year coupon exposures or on measurements of environmental parameters to estimate time of wetness (TOW), sulphur dioxide concentration or deposition rate, and sodium chloride deposition rate. ISO 9224 provided an approach to calculating the extent of corrosion damage from extended exposures for five types of engineering metals based on application of guiding corrosion values (average and steady-state corrosion rates) for each corrosivity categories in ISO 9223. ISO 9225 provided the measurements techniques for the sulphur dioxide concentration or deposition rate, and sodium chloride deposition rate, needed as classification criteria in ISO 9223. ISO 9226 provided the procedure for obtaining one-year atmospheric corrosion measurements on standard coupons.
- **MICAT cooperative programme: “Ibero-American Atmospheric Corrosivity Map”** [7]. The MICAT programme was launched in 1988 as part of the Ibero-American CYTED “Science and Technology for Development” international programme and ended after six years of activities. Fourteen countries participated in the programme, whose goals were: (i) to obtain a greater knowledge of atmospheric corrosion mechanisms in the different environments of Ibero-America; (ii) to establish, by means of suitable statistical analysis of the results obtained, mathematical models that allow the calculation of atmospheric corrosion as a function of climate and pollution parameters; and (iii) to elaborate atmospheric corrosivity maps of the Ibero-American region.
- **ICP/UNECE cooperative programme** [8]. Airborne acidifying pollutants are known to be one of the major causes of corrosion of different materials, including the extensive damage that has been observed on historic and cultural monuments. In order to fill some important gaps in the knowledge of this field, the Executive Body for the Convention on Long-Range Transboundary Air Pollution (CLRTAP) decided to launch an International Cooperative Programme within the United Nations Economic Commission for Europe (ICP/UNECE). The programme started in September 1987 and initially involved exposure at 39 test sites in 11 European countries and in the United States and Canada. The aim of the programme was to perform a quantitative evaluation of the effect of sulphur pollutants in combination with NO*x* and other pollutants as well as climatic parameters on the atmospheric corrosion of important materials.

**Figure 1:**
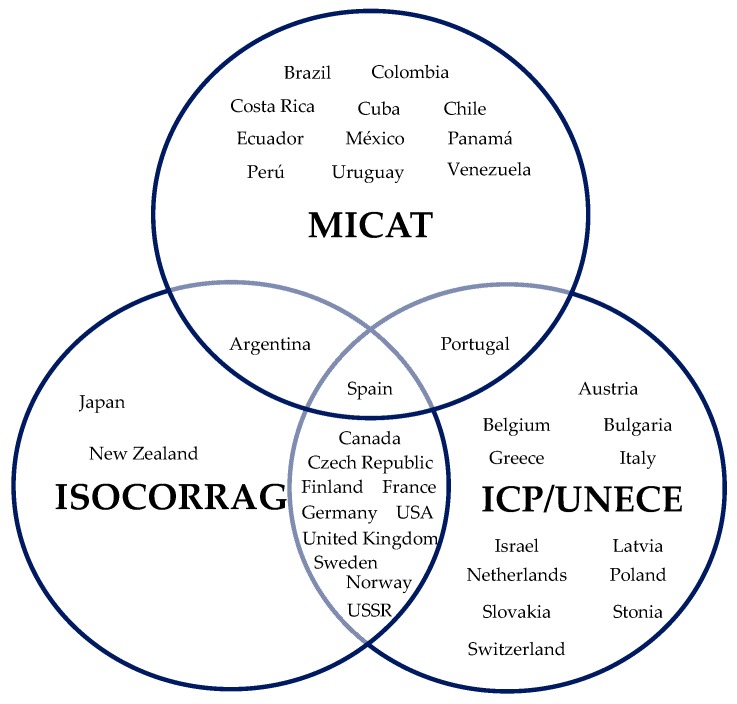
International Collaborative programmes on atmospheric corrosion and participant countries.

Figure 1 shows the countries participating in each one of these programmes. The atmospheric corrosion stations are basically located in Europe, America and Asia, covering a broad range of meteorological and pollution conditions.

**Figure 2:**
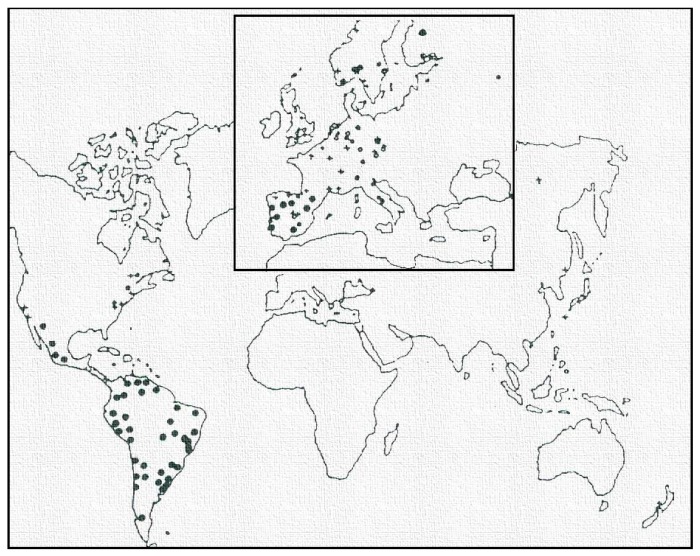
Atmospheric corrosion stations networks: ISOCORRAG (+), ICP (°) and MICAT (●) [12].

Though the three programmes, ISOCORRAG, ICP/UNECE and MICAT, each have their own particular characteristics, they nevertheless share a number of common objectives. The similarity of certain aspects of their methodologies allows a welcome meeting point between the three programmes, as was suggested by Morcillo in the 11th International Corrosion Congress held in Florence in April 1990, in the session on atmospheric corrosion where the three cooperative programmes were presented [9,10,11]. Such a meeting point would allow, for the first time, a worldwide perspective (38 countries) on the problem of atmospheric corrosion, covering a broad spectrum of climatological and atmospheric pollution conditions, never before considered in the abundant published literature on atmospheric corrosion. This idea was taken up at UNECE (Figure 2) by the “Working Group on Effects” of Executive Body for the Convention on Long-Range Transboundary Air Pollution [12].

The statistical analysis of data obtained in atmospheric corrosion studies in order to obtain correlation equations that allow the estimation of annual corrosion rates from meteorological and pollution parameters is a matter of great interest. Such equations are known as damage or dose/response functions. They often incorporate the SO_2_ concentration, the chloride concentration in areas close to the sea, and a parameter representing the wetness of the metallic surface (relative humidity, number of days of rain per year, time of wetness, etc.). Models for predicting the corrosion damage of metals in the atmosphere are useful when it comes to answering questions on the durability of metallic structures, determining the economic costs of damage associated with the degradation of materials, or acquiring knowledge about the effect of environmental variables on corrosion kinetics.

Abundant literature has been published on these models and damage functions. For instance, for long-term prediction of carbon steel atmospheric corrosion, mention may be made of the work of Benarie and Lipfert [13], Pourbaix et al. [14], Feliu et al. [15,16], Knotková and Barton [17], Kucera [18], Mc Cuen and Albrecht [19], Albrecht and Hall [20], Panchenko et al. [21,22], Melchers [23,24], etc. Recent reviews on corrosion models for long-term prediction of atmospheric corrosion has been made by Morcillo et al. [25] and Adikari and Munasinghe [26].

The purpose of this work is to bring together the three databases from the three international cooperative programmes (ICP/UNECE, MICAT and ISOCORRAG), carrying out a statistical analysis of the results they contain in order to establish mathematical expressions which allow an estimation of the extent of atmospheric corrosion of carbon steel during first-year exposure as a function of meteorological and pollution parameters.

## 2. Experimental

### 2.1. ICP/UNECE Programme

Twenty-four countries participated in the exposure programme with a total of 55 exposure sites. These sites included industrial, urban and rural atmospheres. Marine atmosphere exposures were not included. A list of the sites together with their code is given in Table 1.

Exposure always started in the autumn, typically from October of one year to September of the following year. The test site network originally consisted of 39 sites, which were all part of the original eight-year exposure between 1987 and 1995. Subsequently, in a four-year exposure programme carried out between 1997 and 2001, only part of the original sites were kept and eight new test sites were added. Since then, new sites have joined. Compared to the 2008–2009 exposure, the sites Lahemaa and Lincoln were withdrawn from the 2011 to 2012 exposure while a new site in St Petersburg (Russia) was added. In the 2014–2015 exposure, two new test sites, Hameenlina (Finland) and Zilina (Slovakia), were included.

**Figure 3:**
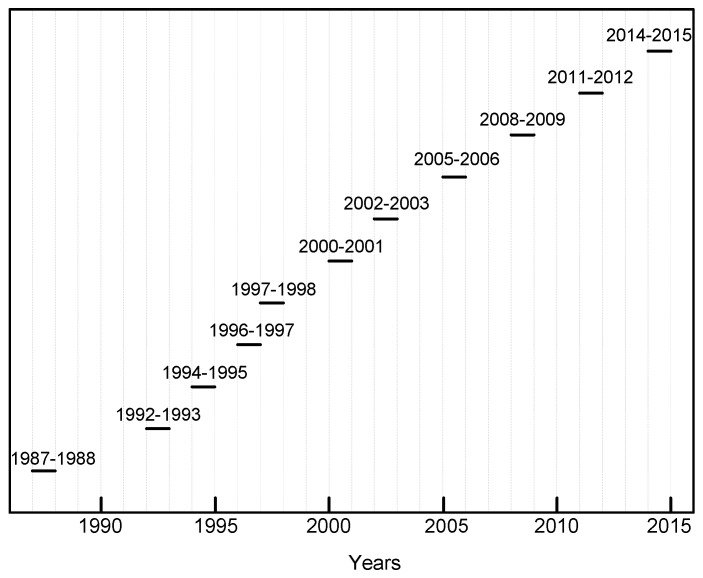
ICP/UNECE programme: Diagram showing the exposure sequences.

Figure 3 shows a diagram of the exposure schedule. For each exposure and site, three identical flat samples were exposed. Average corrosion values for these three panels were obtained. A detailed description of the material and methods for measuring environmental parameters and the evaluation of corrosion attack is provided in Reference [8].

### 2.2. ISOCORRAG Programme

Fourteen countries participated in the exposure programme with a total of 53 exposure sites. These sites included industrial, urban, rural, marine and costal locations in temperate, tropical and arctic zones. A list of the sites together with their code is given in Table 2.

Flat carbon steel specimens were exposed in triplicate, fixing their size and thickness in accordance with the provisions of standard ISO 8565 [27]. A detailed description of the exposed material is provided in Reference [1].

**Figure 4:**
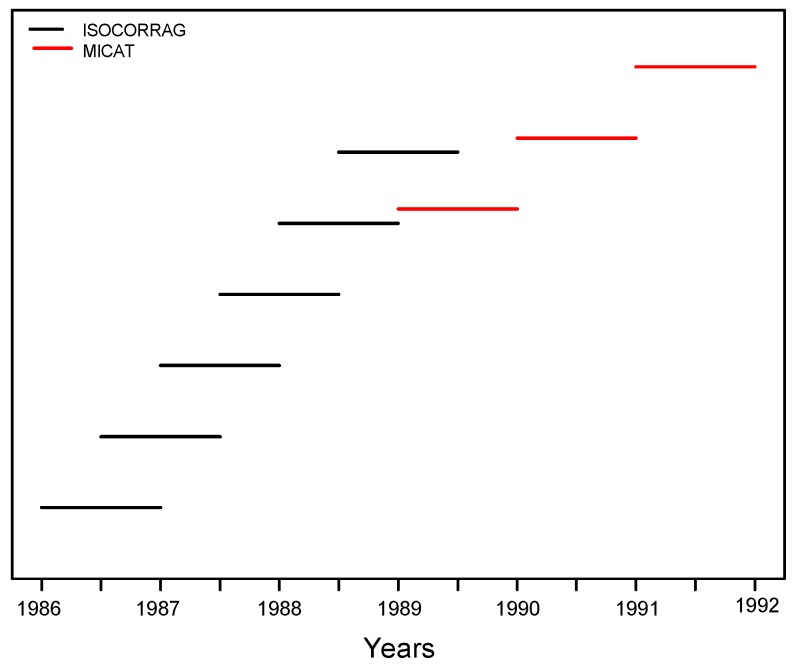
ISOCORRAG and MICAT programmes: diagrams showing the exposure sequences.

A set of specimens was initially exposed for one-year exposure at each site. After six months, another set of specimens was exposed for one-year exposure. After one year, the first set of one-year exposed specimens was removed and another set of one-year specimens was exposed. Every six months, this process was repeated until six sets of specimens had been exposed for one year. Figure 4 shows a diagram of the exposure schedule. The original exposure was planned to begin in the autumn of 1986, but several delays occurred at various sites.

A detailed description of the material and methods for measuring environmental parameters and the evaluation of corrosion attack is provided in Reference [1].

### 2.3. MICAT Programme

Fourteen countries participated in the exposure programme with a total of 75 exposure sites. These sites included industrial, urban, rural and marine atmospheres. A list of the sites together with their code is given in Table 3.

Flat carbon steel panels were exposed in triplicate. A detailed description of the exposed material can be found in the book published with all the results of the project [7]. Figure 4 shows a diagram of the exposure schedule. The original exposure was planned to begin in 1989, but several delays occurred at various sites.

A detailed description of the material and methods for measuring environmental parameters and the evaluation of corrosion attack is provided in Reference [7].

### 2.4. Analysis of Data Properties

Before statistically analysing all the data collected from the various sources (data mining), data screening has been carried out. There follows a description of the criteria governing this screening:

1. Extremely cold stations, with annual average temperatures below 0 °C, have been removed from the statistical analysis. Such is the case of the stations at Svanvik (Norway), Murmansk and Ojmjakon (USSR), Jubany (Argentina), Marsch (Chile) and Artigas (Uruguay), the latter three being Antarctic scientific bases. Low temperatures cause the metallic surface to be covered with an ice layer for long time periods during the year, considerably impeding the development of corrosion processes. This ice layer reduces oxygen access to the metallic surface and its time of wetness, decreasing corrosion rates to extremely low values [28,29,30,31].
2. In stations characterised as rural environments where SO_2_ and Cl^−^ deposition rates have not been determined due to being insignificant, values have been estimated for both pollutants. The figures indicated in Table 4, Table 5 and Table 6 correspond to the average value of the 0–3 mg Cl^−^/m^2^.d range (level S_0_) and the 0–4 mg SO_2_/m^2^.d range (level P_0_) according to standard ISO 9223 [3]. In those cases where both pollutants have been estimated, an average of the corrosion data from available annual series has been made.
3. For test stations located in non-rural environments, all corresponding annual series data, or even the entirety of the available information, have been removed in those cases where, for some reason, meteorological or pollution data are not included.
4. Chloride ion pollution data have not been determined for stations in the ICP/UNECE programme, which only considers non-marine test sites, unlike the other two exposure programmes (ISOCORRAG and MICAT). Therefore, the annual corrosion rate data and meteorological and SO_2_ deposition rate obtained are only included in the statistical analysis for non-marine environments. In this respect, the criteria adopted has been to remove from the ICP/UNECE database all stations located at a distance of less than 2 km from the seashore, supposing in these cases a chloride ion deposition level of more than 3 mg/m^2^.d (lower level S_1_ according to standard ISO 9223 [3]). Bilbao station (Spain), despite being characterised by high SO_2_ values, has been removed because of its location very close to the port.

**Figure 5:**
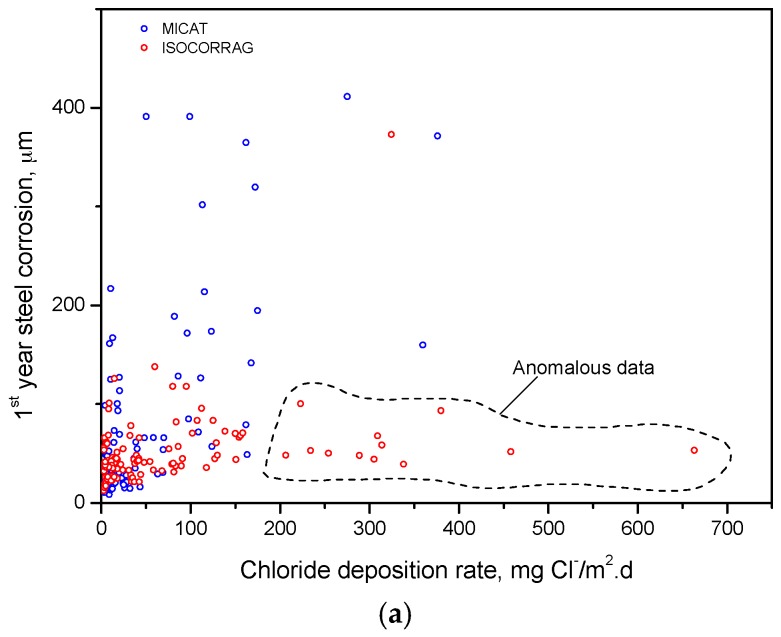

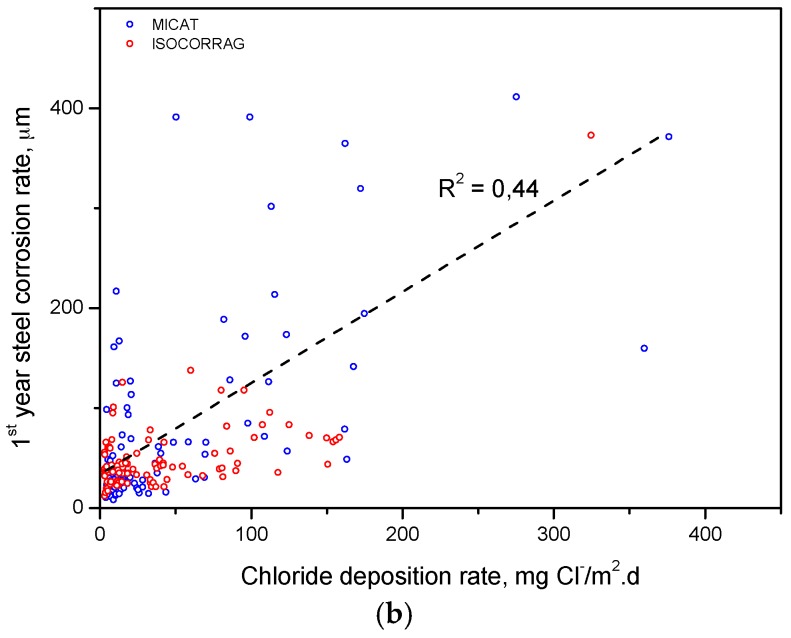
Relationship between annual steel corrosion and chloride deposition rate in marine test sites including in MICAT and ISOCORRAG databases (**a**); and the same relationship after anomalous data were eliminated (**b**).

On the other hand, a series of anomalous values have been observed at stations characterised as marine environments (ISOCORRAG and MICAT databases). Figure 5a shows the relationship between the variables of corrosion (µm/y) and salinity (mg Cl^−^/m^2^.d) in both databases. In this figure it is possible to see a cloud of points with very high salinity values (above 200 mg Cl^−^/m^2^.d) which does not seem to agree with the relatively low carbon steel corrosion values found (50–100 µm). It is also seen that a considerable rise in the marine chloride deposition rate (from 200 to 650 mg Cl^−^/m^2^.d) does not result in greater first-year corrosion of carbon steel, which is contradictory to the abundant literature on this matter recently reviewed by Alcántara et al. [32].These data have therefore been considered to be anomalous, and have been removed from the database. The corrosion stations removed for this reason are: Saint Remy (France), Tannanger (Norway) and Kvarnvik (Sweden). Figure 5b shows the linear relationship between these two variables after removing the aforementioned testing stations.

### 2.5. Integration of ICP/UNECE, ISOCORRAG and MICAT Databases

Table 4, Table 5 and Table 6 present the databases finally considered for the ICP/UNECE, ISOCORRAG and MICAT programmes for statistical analysis after the screening mentioned in the preceding section.

ICP/UNECE: Table 4 presents the corrosion data obtained at the different testing stations along with the corresponding annual average values for the meteorological and pollution parameters measured in the programme: temperature (°C), precipitation (mm/y), relative humidity (%) and SO_2_ deposition rate (mg/m^2^.d).

ISOCORRAG: Table 5 presents the corrosion data obtained at the different testing stations along with the corresponding annual average values for the meteorological and pollution parameters measured in the programme: temperature (°C), relative humidity (%) (only for stations in the Czech Republic), time of wetness (annual fraction), SO_2_ deposition rate (mg/m^2^.d) and chloride ion deposition rate (mg/m^2^.d).

MICAT: Table 6 presents the average corrosion value obtained at the different testing stations along with the annual average values for the meteorological and pollution parameters measured in the programme: temperature (°C), relative humidity (%), time of wetness (annual fraction), precipitation (mm/y), SO_2_ deposition rate (mg/m^2^.d) and chloride ion deposition rate (mg/m^2^.d).

There follows an indication of the similarities and differences between the experimental methods used in the three collaborative programmes and how this has affected the integration of the three databases for statistical analysis:

(a) Evaluation of the first-year corrosion (mass loss) of carbon steel according to ISO 9226 [6].
(b) Measurement of meteorological parameters (T, RH, and precipitation) according to standard conventional procedures. The ISOCORRAG programme does not consider precipitation or RH (except at the Czech Republic stations).
(c) Estimation of TOW according to ISO 9223 [2,3]. The ICP/UNECE programme does not consider this parameter.
(d) Measurement of SO_2_ deposition rate according to ISO 9225 [5].
(e) Measurement of Cl deposition rate according to ISO 9225 [5]. The ICP/UNECE programme does not consider marine atmospheres.

## 3. Discussion

It is a well known fact that the atmospheric corrosion of metals is influenced by many factors: (a) external conditions, meteorology and air pollution; (b) exposure conditions; (c) construction conditions; (d) internal conditions, such as nature of the metal and characteristics of corrosion products; among others.

Over the years, many models have been developed to assess the corrosion of carbon steel in the atmosphere. The specialised literature offers a large range of damage functions that relate atmospheric corrosion of carbon steel with environmental data. However, most of them are of limited applicability as they were obtained with minimal variations in meteorological parameters (small geographic areas). Special mention should be made of the efforts of Benarie and Lipfert [13] to develop universal corrosion functions in terms of atmospheric pollutants, meteorological parameters and the rain pH, as well as the work of Feliu et al. [15] compiling a comprehensive literature survey of worldwide atmospheric corrosion and environmental data that were statistically processed to establish general corrosion damage functions in terms of simple meteorological and pollution parameters. Reviews on this subject can be found in [7,26].

In recent decades, several international exposure programmes (ISOCORRAG [1], ICP/UNECE [8], and MICAT [7]) have been carried out with the aim of more systematically obtaining relationships (dose/response functions) between atmospheric corrosion rates and pollution levels in combination with climate parameters. Integration of the databases obtained in these three exposure programmes may make it possible to obtain universal damage functions based on a worldwide variety of meteorological and pollution conditions. This has been the chief aim of the work reported here.

The data have been fitted to the following linear equation:

C = a_1_ + a_2_ RH + a_3_ P + a_4_ T + a_5_ TOW + a_6_ SO_2_ + a_7_ Cl, (1)

This equation is quite simple. Other combinations between the different variables or other more sophisticated statistical treatments would likely yield better fits, but the aim of this work has been to use as simple as possible a relation.

According to this model, the dependent variable C (carbon steel annual corrosion in µm) is interpreted as a linear combination of a set of independent variables: RH, annual average relative humidity, in per cent; T, annual average temperature, in °C; P, annual precipitation, in mm; TOW, time of wetness, annual fraction of number of hours/year in which RH > 80% and T > 0 °C [3]; SO_2_, SO_2_ pollution, in mg/m^2^.day; and Cl, chloride pollution, in mg/m^2^.day. Each independent variable is accompanied by a coefficient (a_2_–a_7_) which indicates the relative weight of that variable in the equation. The equation also includes a constant a_1_.

The minimum-quadratic regression equation is constructed by estimating the values of coefficients a_1_–a_7_ from the regression model. These estimates are obtained trying to keep the squared differences between the values observed and the forecast values to a minimum. In order to know the model’s fitting quality in relation to the experimental data, the statistic R^2^ is used, i.e., the square of the multiple correlation coefficient. R^2^ expresses the proportion of variance of the dependent variable which is explained by the independent variables.

There are different methods to select the independent variables that a regression model must include. The most widely accepted is the stepwise regression model. With this method, the best variable is firstly selected (always with a statistical criterion); then the best of the rest is taken; and so on, until no variables that fulfil the selection criteria remain. A great change in R^2^ when a new variable is inserted in the equation indicates that this variable provides unique information on the dependent variable that is not supplied by the other independent variables.

The study has been carried out with the assistance of a commercial computer programme (SSPS) [33]. Statistical processing has been carried out considering marine and non-marine atmospheres separately. The variability of the corrosion and environmental data is shown in Table 7.

### 3.1. Non-Marine Atmospheres

The three databases have been analysed together: ICP/UNECE database (Table 4) (all corrosion stations), and ISOCORRAG (Table 5) and MICAT (Table 6) databases (only those stations with a chloride ion deposition rate of Cl^−^ < 3 mg/m^2^.d).

The meteorological and pollution parameters common to all three databases and which have been included in the treatment are: temperature, relative humidity and SO_2_ pollution, though relative humidity is only available in the ISOCORRAG database for stations in the Czech Republic [34]. The stepwise method has been used to select what independent variables are included in the treatment and which are significant. Statistically all the variables are significant, with SO_2_ being the variable that contributes with an extremely high percentage (R^2^ = 0.671) in the total recorded variance (R^2^ = 0.725). RH and T also contribute, raising the R^2^ by 0.037 and 0.017 units, respectively.

**Figure 6:**
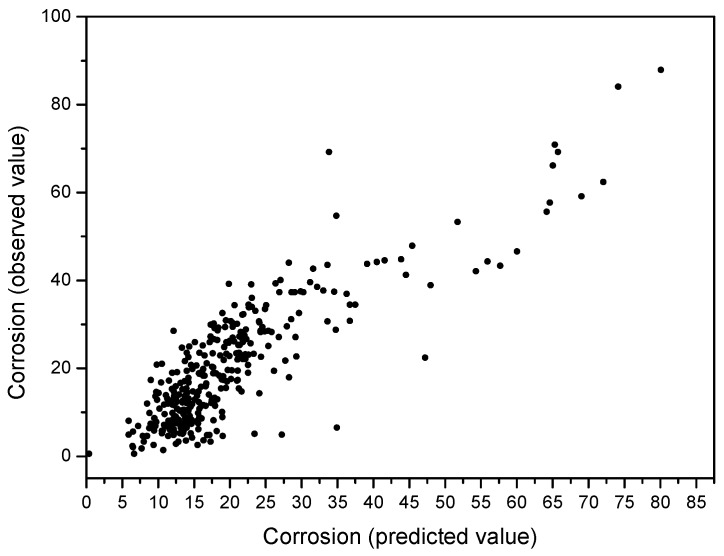
Relationship between predicted and observed carbon steel corrosion values by applying Equation (2).

The resulting regression equation is:

C = −26.32 + 0.43 T + 0.45 RH + 0.82 SO_2_; (R^2^ = 0.725) (N = 333), (2)

where N is the number of data. The model explains 72.5% of the dependent variable. This is the regression equation with non-standard coefficients, partial regression coefficients which define the regression equation at direct scores. Figure 6 shows the relationship between predicted and observed carbon steel corrosion values by applying Equation (2).

The standardised partial regression coefficients are the coefficients that define the regression equation when it is obtained after standardising the original variables, i.e., after converting the direct scores into typical scores. These coefficients make it possible to evaluate the relative importance of each independent variable within the equation. The regression equation with standardised coefficients is shown in Equation (3):

C = 0.15 T + 0.26 RH + 0.82 SO_2_; (R^2^ = 0.725) (N = 333), (3)

Great caution must be used when making corrosion predictions with independent variable values that are much larger or smaller than those used to derive these equations (see Table 7).

The goodness of the fit is slightly higher than the damage function developed by ICP/UNECE for this type of atmospheres using a more sophisticated mathematical model (Table 8), with a notably lower number of data (N = 148) used in the statistical treatment.

If, instead of RH, time of wetness (TOW) were to be considered (No. of hours in which RH > 80% and T > 0 °C, and therefore less precise than RH), taking into consideration the ISOCORRAG and MICAT databases the resulting regression equation would be:

C = 6.58 + 0.75 SO_2_ + 20.85 TOW; (R^2^ = 0.684) (N = 138), (4)

with a slightly lower regression coefficient than Equation (2). In this case, the temperature is a non-significant variable and the greatest specific weight again corresponds to SO_2_, which contributes to the total recorded variance with an R^2^ = 0.646 of a total R^2^ = 0.684.

Considering another related parameter, such as precipitation (P) (in mm/y), instead of RH, the following regression equation would be obtained:

C = −29.26 + 0.87 SO_2_ + 0.51 RH + 0.49 T − 0.003 P; (R^2^ = 0.625) (N = 315), (5)

in which the resulting regression coefficient decreases even more.

### 3.2. Marine Atmospheres

As in the previous case, the stepwise method has been used to select what independent variables are included in the statistical treatment and are significant. In this case, given that the ICP/UNECE programme did not measure the Cl ion deposition rate as it only considered non-marine testing stations, only stations from the ISOCORRAG and MICAT databases have been considered, taking as independent variables the chloride deposition rate (Cl), SO_2_ pollution, temperature and time of wetness. As has been noted above, data on relative humidity are not available for ISOCORRAG stations.

In this case Cl is the variable that contributes with the highest percentage (R^2^ = 0.411) to the total recorded variance (R^2^ = 0.474). SO_2_ also contributes, raising the R^2^ by 0.041 units. TOW is also a significant variable but raises the R^2^ by only 0.022 units. Temperature is excluded as a significant variable.

The resulting regression equation is:

C = −24.50 + 0.75 Cl + 0.67 SO_2_ + 77.32 TOW (R^2^ = 0.474) (N = 206), (6)

while in standard coefficients the resulting equation would be:

C = 0.62 Cl + 0.22 SO_2_ + 0.15 TOW (R^2^ = 0.474) (N = 206), (7)

**Figure 7:**
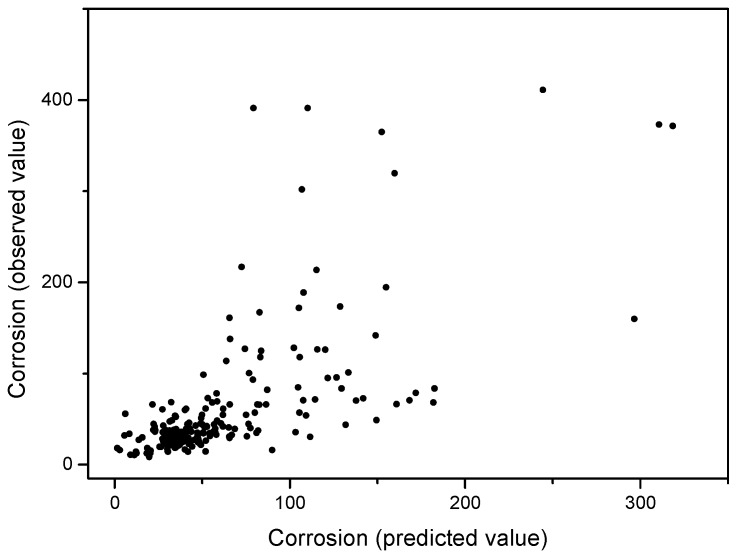
Relationship between predicted and observed carbon steel corrosion values by applying Equation (6).

Cl and SO_2_, in this order, are the variables with the greatest weight in the carbon steel corrosion rate. Figure 7 shows the relationship between predicted and observed carbon steel corrosion values by applying Equation (6).

The goodness of this fit is slightly lower than in the MICAT programme and notably lower than those obtained in the ISOCORRAG programme (Table 8). On the other hand, the number of data used in the statistical treatment to obtain the damage function (Equations (6) and (7)) is somewhat higher.

Replacing the TOW variable with RH, information that is available in MICAT and perhaps in ISOCORRAG records, could possibly have improved the fitting quality achieved.

It would be also helpful to make new fits using more sophisticated mathematical models, and we encourage experts in statistics to do this. For this purpose, a good starting point could be the perfected broad database that are presented in this work (Table 4, Table 5 and Table 6).

### 3.3. Contribution of the Information Supplied for Each International Programme

Having established the integrated database, it is of interest to compare the damage functions and correlation coefficients obtained using the combined information of the three programmes (Equations (3) and (7)) with those obtained separately using the information supplied each individual programme. In this way, it may be possible to determine the contribution of each programme to the general damage functions. The results obtained with this treatment of the information are shown in Table 9.

Considering non-marine atmospheres, it is seen that the greatest volume of information (81%) is supplied by the ICP/UNECE programme, which establishes SO_2_, RH and T as the most significant variables, the former with the greatest weight. The contribution of the ISOCORRAG programme (R^2^ = 0.867), which incorporates a relatively small amount of data as only the Czech Republic testing stations supplied information on these three variables, makes only a slight improvement to the general correlation coefficient (0.725) in relation to R^2^ supplied by the ICP/UNECE programme (0.674). With regard to the information provided by the MICAT programme, the low correlation coefficient obtained (0.398) may be an indication of poorer data quality than in the other programmes, perhaps due to the participation in MICAT of countries with little or no prior experience in the field of atmospheric corrosion [7]. In this respect, it should not be overlooked that one of the MICAT research programme’s main aims was precisely to promote the development of this line of research in some of the participating countries [11].

Considering marine atmospheres, information has been supplied only by the ISOCORRAG and MICAT programmes, with a similar amount of data in each case. These data show the important effect of Cl and SO_2_ pollutants (in this order) on the magnitude of the atmospheric corrosion of carbon steel. The greatest contribution to the general damage function seems to correspond to the ISOCORRAG programme, which includes TOW as an also significant variable. The joining of data from the two programmes, whose individual damage functions have only low correlation coefficients, yields a combined damage function with an even lower correlation coefficient.

## 4. Goodness of the Fits

As we have seen, the environmental parameters considered in this work only partly explain the corrosion data. The goodness of fit of experimental data to a proposed model is often measured by the statistical R^2^, i.e., the square of the correlation coefficient (R) between the observed values of the dependent variable and those predicted from the fitted line.

In previous work [15,16], the authors noted a series of causes that may affect the goodness of the fit obtained:

- Oversimplification of the mathematical model. In this sense, the best fits obtained in the ISOCORRAG programme, including or excluding the MICAT databases and data from Russian sites in frigid regions [1] (see Table 8), may have been at least partly due to considering interactions between the meteorological and pollution variables. One example of complex interactions involves the RH (or TOW), which in addition to its effect on the wetting of the metal surface, plays a major role in the mechanisms whereby air pollutants take part in corrosion.
- The lack of quality in corrosion and environmental data.
- Probable occurrence of other variables with marked effects on corrosion that were not considered in the statistical treatment. For instance, besides sulphur dioxide and chlorides, other pollutants not considered in the study may have played an important role in the corrosion data. In this sense, mention should be made of the effort made by ICP/UNECE to consider in the new damage functions (for the multi-pollutant situation) other important pollutants in terms of their effect on the corrosion of weathering steel [18].
- Many effects that have not been considered. To mention just a few: The magnitude of diurnal and seasonal changes in meteorological and pollution parameters, the frequency, duration and type of wetting and drying cycles, and the time of year when exposure is initiated.

Finally, it would be desirable, as noted by Leygraf et al. [36], to develop models on the basis of mechanistic considerations instead of statistical considerations, and recent work has attempted to take this into account [37,38,39]. Nevertheless, there is still a very long way to go to reach this desired goal. As Roberge et al. [40] note, the results obtained with mechanistic models reveal why statistical schemes have only a limited accuracy. There are many variables that can change from site to site which are not accounted for in the standard set of environmental variables. According to this researcher, a single transferable and comprehensive environmental corrosivity prediction model is yet to be published, and may ultimately not be possible due to the complexity of the issues involved.

## 5. Conclusions

The following may be considered the most relevant conclusions of this study:

- A highly complete and perfected database has been obtained from published data from the ISOCORRAG, ICP/UNECE and MICAT programmes.
- The number of data used in the statistical treatment has been much higher than that used in other damage functions previously published by the different programmes.
- The statistical treatment carried out has differentiated between two types of atmospheres: non-marine and marine, which may represent a significant simplification for persons with little knowledge of the atmospheric corrosion process who wish to estimate the corrosion of carbon steel exposed at a given location. Moreover, having considered a highly simple polynomial function (Equation (2)) in the work may also be an advantage in this sense.
- With regard to non-marine atmospheres, by joining the three databases (ISOCORRAG, ICP/UNECE and MICAT), the following damage function has been obtained:
  C = −26.32 + 0.43 T + 0.45 RH + 0.82 SO_2_ (R^2^ = 0.725) (N = 333), where SO_2_ is the variable of greatest significance. The inclusion of TOW (or precipitation) instead of RH leads to lower regression coefficients. The goodness of the fit obtained (R^2^) is slightly higher than that obtained in the ICP/UNECE programme with a more sophisticated function.
- In relation with marine atmospheres, only the ISOCORRAG and MICAT databases have been considered (ICP/UNECE did not consider this type of atmospheres). The damage function obtained is:
  C = −24.50 + 0.75 Cl + 0.67 SO_2_ + 77.32 TOW (R^2^ = 0.474) (N = 206), where Cl and SO_2_, in this order, are the most significant variables. The goodness of the fit is slightly lower than that obtained in the MICAT programme, which uses a similar type of function, and notably lower than that obtained in the ISOCORRAG programme using a more sophisticated type of function.

## Figures and Tables

**Table 1:** Atmospheric corrosion test sites included in the ICP/UNECE Programme.

Code	Country	Test Site	Code	Country	Test Site
P01	Czech Republic	Praha	P29	United Kingdom	Clatteringshaws Loch
P02		Kasperske Hory	P30		Stoke Orchard
P03		Kopisty	P31	Spain	Madrid
P04	Finland	Espoo	P32		Bilbao
P05		Ahtari	P33		Toledo
P06		Helsinki	P34	Russia	Moscow
P07	Germany	Waldhof-Langenbrugge	P35	Estonia	Lahemaa
P08		Aschaffenburg	P36	Portugal	Lisbon-Jeronimo Mon.
P09		Langenfeld-Reusrath	P37	Canada	Dorset
P10		Bottrop	P38	USA	Steubenville
P11		Essen-Leithe	P39		Res. Triangle Park
P12		Garmisch-Partenkirchen	P40	France	Paris
P13	Italy	Rome	P41	Germany	Berlin
P14		Casaccia	P43	Israel	Tel Aviv
P15		Milan	P44	Norway	Svanvik
P16		Venice	P45	Switzerland	Chaumont
P17	Netherlands	Vlaardingen	P46	United Kingdom	London
P18		Eibergen	P47	USA	Los Angeles
P19		Vredepeel	P49	Belgium	Anvterps
P20		Wijnandsrade	P50	Poland	Katowice
P21	Norway	Oslo	P51	Greece	Athens
P22		Borregaard	P52	Latvia	Riga
P23		Birkenes	P53	Austria	Vienna
P24	Sweden	Stockholm S	P54	Bulgaria	Sophia
P25		Stockholm C	P55	Russia	St Petersburg
P26		Aspvreten	P57	Finland	Hameelina
P27	United Kingdom	Lincoln Catch.	P59	Slovakia	Zilina
P28		Wells. Catch.			

**Table 2:** Atmospheric corrosion test sites included in the ISOCORRAG Programme.

Code	Country	Test Site	Code	Country	Test Site
I01	Argentina	Iguazu	I29	Norway	Birkenes
I02		Camet	I30		Tannanger
I03		Buenos Aires	I31		Bergen
I04		San Juan	I32		Svanvik
I05		Jubany Base	I33	Spain	Madrid
I06	Canada	Bourcherville	I34		El Pardo
I07	Czech Republic	Kasperske Hory	I35		Lagoas-Vigo
I08		Praha-Bechovice	I36		Baracaldo, Vizcaya
I09		Kopisty	I37	Sweden	Stockholm-Vanadis
I10	Germany	Bergisch Gladbach	I38		Bohus Malmon, Kattesand
I11	Finland	Helsinki	I39		Bohus Malmon, Kvarnvik
I12		Otaniemi	I40	United Kingdom	Stratford, East London
I13		Ahtari	I41		Crowthorne, Berkshire
I14	France	Saint Denis	I42		Rye, East Sussex
I15		Ponteau Martigues	I43		Fleet Hall
I16		Picherande	I44	USA	Kure Beach, N. Carolina
I17		Saint Remy	I45		Newark-Kerney, New Jersey
I18		Salins de Giraud	I46		Panama Fort Sherman Costal Site
I19		Ostende, Belgium	I47		Research Triangle Park, N. Carolina
I20		Paris	I48		Point Reyes, California
I21		Auby	I49		Los Angeles, California
I22		Biarritz	I50	USSR	Mursmank
I23	Japan	Choshi	I51		Batumi
I24		Tokyo	I52		Vladivostok
I25		Okinawa	I53		Ojmjakon
I26	New Zealand	Judgeford, Wellington			
I27	Norway	Oslo			
I28		Borregaard			

**Table 3:** Atmospheric corrosion test sites included in the MICAT Programme.

Code	Country	Test Site	Code	Country	Test Site
M01	Argentina	Camet	M38	Ecuador	Esmeraldas
M02		Villa Martelli	M39		San Cristóbal
M03		Iguazú	M40	Spain	León
M04		San Juan	M41		El Pardo
M05		Jubany	M42		Barcelona
M06		La Plata	M43		Tortosa
M07	Brazil	Caratinga	M44		Granada
M08		Ipatinga	M45		Lagoas-Vigo
M09		Arraial do Cabo	M46		Labastida
M10		Cubatão	M47		Arties
M11		Ubatuba	M48	México	Mexico
M12		São Paulo	M49		Cuernavaca
M13		Río de Janeiro	M50		San Luis Potosí
M14		Belem	M51		Acapulco
M14		Fortaleza	M52	Panamá	Panamá
M16		Brasilia	M53		Colon
M17		Paulo Afonso	M54		Veraguas
M18		Porto Velho	M55		Chiriquí
M19	Colombia	Isla Naval	M56	Perú	Piura
M20		San Pedro	M57		Villa Salvador
M21		Cotové	M58		San Borja
M22	Costa Rica	Puntarenas	M59		Arequipa
M23		Limón	M60		Cuzco
M24		Arenal	M61		Pucallpa
M25		Sabanilla	M62	Portugal	Leixões
M26	Cuba	Ciq	M63		Sines
M27		Cojímar	M64		Pego
M28		Bauta	M65	Uruguay	Trinidad
M29	Chile	Cerrillos	M66		Prado
M30		Valparaíso	M67		Melo
M31		Idiem	M68		Artigas
M32		Petrox	M69		Punta del Este
M33		Marsh	M70	Venezuela	Tablazo
M34		Isla de Pascua	M71		Punto Fijo
M35	Ecuador	Guayaquil	M72		Coro
M36		Riobamba	M73		Matanzas
M37		Salinas	M74		Barcelona, V

**Table 4:** ICP/UNECE data considered in the study.

Code	1st Year Corrosion, µm	T, °C	RH, %	SO_2_ Deposition Rate mg/m^2^.d	Precipitation, mm/y	Code	1st Year Corrosion, µm	T, °C	RH, %	SO_2_ Deposition Rate, mg/m^2^.d	Precipitation, mm/y
P01	55.6	9.5	79	62	639	P23	12.09	6.8	77	0.16	1544
P01	34.48	9.1	73	32.96	684	P23	5.34	6.5	82	0.16	2195
P01	30.66	9.8	77	25.68	581	P24	33.97	7.6	78	13.44	531
P01	29.52	8.6	78	18.88	475	P24	15.27	7	70	4.56	577
P01	23.16	9.9	76	12.24	522	P24	13.1	7.5	73	3.36	581
P01	17.56	9.5	79	7.04	601	P24	13.61	7.4	68	2.64	556
P01	13.1	9.3	72	5.12	513	P24	15.9	6.7	76	2.08	463
P01	12.98	9.3	74	8.88	491	P24	14.76	8.1	81	1.52	635
P01	7.51	10.1	74	5.2	525	P24	10.31	7.1	80	1.28	384
P01	8.52	10.2	70	5.12	534	P24	11.7	8.9	74	1.44	273
P01	8.4	11	73	3.68	414	P24	7.76	7.8	76	0.64	270
P02	28.5	7	77	15.76	850	P24	5.47	7.8	77	0.72	428
P02	19.47	6.6	73	14.32	921	P24	7.38	8.3	81	0.4	330
P02	18.83	7.2	74	9.76	941	P25	33.46	7.6	78	15.68	531
P03	70.87	9.6	73	66.64	426	P25	13.1	7	70	3.76	577
P03	44.53	8.9	71	39.2	432	P25	12.09	7.5	73	2.72	581
P03	44.78	9.7	75	39.36	513	P26	18.7	6	83	2.64	543
P03	37.28	8.5	73	24.48	431	P26	9.54	6	81	1.04	468
P03	30.41	9.9	76	14.64	420	P26	10.31	6.8	82	0.88	525
P03	28.5	9.2	80	14.32	510	P26	8.78	6.5	83	0.64	409
P03	23.41	8.7	73	8.96	463	P26	7.89	5.9	86	0.48	479
P03	23.28	8.3	76	14.48	442	P26	8.78	7.2	86	0.48	772
P03	20.74	9.3	80	10.8	521	P26	5.09	5.6	82	0.48	562
P03	28.24	9.6	79	15.2	417	P26	5.22	6.3	84	0.48	435
P03	26.59	10.8	71	9.12	433	P26	3.56	7.1	82	0.32	452
P04	34.48	5.9	76	14.88	626	P26	2.54	6.7	86	0.32	511
P04	16.67	5.6	79	1.84	755	P26	14.89	7.2	82	0.24	784
P04	15.39	6	80	2.08	698	P27	40.08	9.2	84	14.16	365
P05	16.79	3.1	78	5.04	801	P27	39.31	9.6	82	14.24	530
P05	6.11	3.4	81	0.72	610	P27	30.15	10.5	78	5.44	515
P05	7.51	3.9	83	0.64	675	P27	34.35	10.2	81	7.5	708
P05	6.87	3.2	76	0.48	618	P27	24.81	9.7	81	6	831
P05	6.74	3.5	80	0.72	742	P27	22.9	10.4	78	3.92	548
P05	6.49	4.8	82	0.64	845	P28	32.19	10.8	86	5.76	447
P05	4.83	4.5	80	0.64	713	P28	25.95	10.5	82	2.56	614
P06	34.35	6.3	78	16.56	673	P28	25.19	11.2	79	2.64	696
P06	20.74	6.2	78	3.84	702	P30	39.06	10.2	78	12	610
P06	24.94	6.6	76	4.4	649	P30	29.26	10.3	76	7.44	549
P07	33.84	9.3	80	10.96	631	P31	28.24	14.1	66	14.72	398
P07	29.39	8.9	81	6.56	624	P31	20.61	14.3	67	6.56	360
P07	21.12	9.5	81	3.12	596	P31	19.21	15.7	68	6.24	224
P07	19.85	8.9	82	2.32	615	P31	20.23	14.8	67	9.12	401
P07	18.32	9.5	83	1.68	786	P31	9.16	12.9	61	9.44	765
P07	18.83	9.4	81	1.84	620	P31	9.8	15	62	0.96	560
P07	10.81	8.8	75	1.76	413	P31	7.38	15.3	60	2.08	447
P08	27.1	12.3	77	18.96	627	P31	5.6	15.3	56	1.28	399
P08	14.76	11.4	64	10.08	561	P31	2.29	15.1	53	2.96	267
P08	17.81	11.6	65	7.68	779	P31	0.51	16.2	43	0.48	283
P09	37.28	10.8	77	19.6	783	P31	2.54	16	63	0.56	303
P09	29.39	10.7	79	13.04	619	P33	5.73	14	64	2.64	785
P09	26.59	11.4	81	8.88	841	P33	3.31	13.4	61	1.36	433
P09	26.21	10	78	8.4	781	P33	4.58	14.8	57	3.36	327
P09	25.95	10.9	80	6.64	930	P33	4.58	14	61	0.88	603
P09	12.85	11.6	79	4	997	P33	6.87	14	59	1.2	872
P09	16.54	11.4	76	4.8	647	P33	5.98	12.2	71	0.96	739
P10	47.84	11.2	75	40.48	874	P33	4.2	12.2	78	0.88	411
P10	44.15	10.3	78	33.28	707	P33	5.73	12.1	69	0.72	689
P10	37.4	11.8	80	24.16	913	P33	1.78	14.7	61	0.32	828
P10	37.66	10.5	79	23.52	806	P33	0.51	15.4	58	0.32	430
P10	39.57	11.5	81	19.68	1044	P33	2.04	12.8	60	0.4	516
P10	37.28	11.7	81	14.32	791	P34	23.03	5.5	73	15.36	575
P10	28.24	11.3	77	13.52	780	P34	17.94	5.7	74	22.96	881
P10	27.48	10.8	81	8.88	663	P34	15.39	5.6	71	13.12	667
P10	28.63	11.1	75	7.44	849	P34	17.18	6.5	74	13.7	838
P10	30.92	11.4	78	7.04	880	P34	17.3	7.4	69	13.7	812
P11	43.51	10.5	79	24.24	713	P34	11.7	5.9	71	3.28	750
P11	37.28	10.1	79	18.32	684	P35	23.54	5.5	83	0.72	448
P11	30.66	10.9	78	12.96	889	P35	13.49	5.4	82	1.1	859
P12	17.56	8	82	7.52	1492	P35	12.09	6.9	81	1.04	668
P12	11.45	7.1	84	2.56	1552	P35	12.21	5	81	1.36	655
P12	10.81	7.4	83	1.92	1503	P35	11.2	5.2	80	3.2	403
P13	22.65	15.4	66	23.52	591	P35	7.38	8.8	81	0.88	640
P13	15.78	18.4	68	4.64	602	P36	28.5	12.1	64	5.44	972
P13	17.05	19.4	65	2.96	1125	P36	39.19	18	62	12.88	545
P13	8.02	17.8	53	0.88	625	P36	25.95	19.1	67	3.76	443
P13	8.02	18	66	0.64	1115	P36	27.23	17.9	63	14.16	252
P14	29.9	14.6	71	6.64	650	P37	18.96	5.5	75	2.64	961
P14	18.83	14.9	76	4.16	717	P37	13.99	4.3	80	1.68	1080
P14	15.9	14.5	74	4.16	742	P37	13.23	5.2	80	2.64	1023
P14	8.65	16.3	63	0.56	600	P37	14.76	7.4	75	1.92	788
P14	10.81	14.5	67	0.16	742	P37	11.96	7.2	76	0.48	964
P14	6.49	15.9	69	2.96	857	P38	27.23	14.6	69	7.68	847
P14	10.31	15.7	71	0.88	585	P38	23.54	15.5	64	8.08	982
P14	14.38	15.5	73	0.96	1114	P38	4.83	15.8	68	7.44	1038
P15	46.56	15.3	72	57.76	1125	P39	22.39	12.3	67	46.48	733
P15	25.06	14.3	69	17.68	1092	P39	36.9	11.8	65	34.48	729
P15	22.01	14.5	69	12.32	1077	P39	6.49	11.8	69	30.64	757
P15	23.41	15.9	71	10.32	932	P40	17.43	13.4	67	11.36	572
P15	11.45	15.1	66	9.84	619	P40	18.19	12.7	74	8.08	731
P15	14.63	14	56	5.92	632	P40	11.96	13.3	69	8.96	490
P15	11.96	15	56	3.84	1179	P40	11.58	12.6	73	5.28	571
P15	5.85	13.9	63	1.76	583	P40	7.51	12.7	70	2.48	427
P15	8.02	15.8	63	3.52	1037	P40	6.11	13.2	70	1.28	382
P16	31.17	14.9	77	16.88	714	P40	8.14	13.2	74	6.2	668
P16	26.97	13.2	82	5.04	500	P41	22.14	8.4	76	13.04	473
P16	26.84	13.5	83	5.92	742	P41	22.77	10.4	77	8.72	486
P16	18.96	14.9	83	6.24	638	P41	22.14	11.1	82	7.84	489
P16	13.23	13.7	79	3.36	795	P41	18.32	11.7	71	6.88	473
P16	8.78	14.5	77	1.44	588	P41	15.52	10.1	88	2.24	348
P16	9.8	15	77	0.96	881	P41	11.58	10	72	2.24	570
P17	43.77	10.5	84	28.24	978	P41	7.38	10.3	77	1.84	473
P17	38.55	10.3	83	20.4	860	P43	41.22	24.6	83	28	485
P17	32.57	11	84	16.4	996	P43	32.57	22	70	5.28	254
P18	32.32	9.9	83	8.08	904	P45	11.83	6.2	77	1.2	1135
P18	25.95	9.5	82	5.92	873	P45	8.52	6.9	77	1.04	1053
P18	18.32	10.3	83	3.76	987	P45	7.38	7.2	80	0.8	1281
P19	36.01	10.3	81	10.4	845	P45	4.58	7.3	75	1.04	1011
P19	30.41	10	82	6.64	749	P45	5.22	6.2	80	0.88	1404
P19	22.9	10.9	83	3.6	829	P45	3.31	6.3	80	0.56	950
P20	33.08	10.3	81	10.96	801	P45	2.8	7	79	0.32	1108
P20	26.08	10.1	81	7.44	680	P46	22.52	12.2	70	4.64	706
P20	21.88	11.1	82	4.64	790	P46	21.63	12.1	69	4.64	907
P21	29.13	7.6	70	11.52	1024	P46	19.08	12.7	66	4.64	494
P21	17.18	7.7	68	4.8	440	P47	17.3	17.4	61	0.48	33
P21	12.85	7.5	69	2.32	680	P49	21.76	11.4	76	18.24	834
P21	12.6	6.8	76	3.2	764	P49	23.54	11.7	75	10.8	993
P21	11.83	6.6	79	3.28	523	P49	13.99	11.9	65	11.04	674
P21	12.34	7.2	75	2.48	1050	P50	34.48	9.4	81	27.52	870
P21	7.12	6.4	74	1.36	794	P50	30.79	8.2	76	30.88	702
P21	9.54	7.2	74	1.04	869	P50	28.75	7.5	76	28.88	674
P21	7.51	6.9	76	1.6	737	P50	28.37	7.7	84	12.24	651
P21	5.22	7.4	74	0.48	715	P50	25.32	8.8	74	12.96	676
P21	7.89	7.5	76	3.36	805	P50	4.58	10.7	71	10.72	484
P22	54.71	6	78	28.64	1116	P51	10.05	18.7	62	11.36	461
P22	44.02	7	76	21.12	628	P51	6.87	18.5	56	3.36	325
P22	42.62	7.4	76	25.04	819	P51	19.85	18.7	62	6.32	570
P23	24.68	6.5	80	1.04	2144	P52	10.56	8.2	77	2.8	633
P23	16.79	5.9	75	0.56	1189	P52	8.02	7.8	75	0.8	589
P23	13.87	6.4	76	0.56	1420	P53	10.43	11.2	73	2	855
P23	14.38	5.6	75	0.32	1182	P53	5.73	11.3	73	2.64	555
P23	12.85	6.2	79	0.16	1744	P53	10.56	12	71	3.36	527
P23	14.5	6.6	83	0.24	2333	P54	8.91	11.5	70	10.8	651
P23	8.27	5.9	81	0.24	1390	P55	12.6	6.1	76	2.48	636
P23	13.61	6.2	79	0.4	1623	P59	13.74	9.7	74	5.2	664
P23	9.8	4.2	81	0.08	1392						

**Table 5:** ISOCORRAG data considered in the study.

Code	1st Year Corrosion, μm	T, °C	RH, %	TOW, Annual Fraction	Deposition Rates, mg/m^2^.d		Code	1st Year Corrosion, μm	T, °C	RH, %	TOW, Annual Fraction	Deposition Rates, mg/m^2^.d	
					SO_2_	Cl						SO_2_	Cl
I01	5.8	22.9		0.615	2	1.5	I25	54.7	24		0.478	8.48	75.85
I02	24.9	14.1		0.682	2	18.21	I25	57.2	23.4		0.439	8.84	86.17
I02	54.8	13.9		0.708	2	24.39	I25	44.8	23.2		0.338	9.76	91.03
I02	78.2	14.3		0.725	2	33.38	I25	39.2	23.5		0.354	10.4	78.89
I02	66	14.5		0.736	2	42.48	I27	26.1	6.7		0.299	13.84	1.21
I02	68.3	14.2		0.711	2	32.16	I27	26.6	6.2		0.326	11.84	2.18
I03	14.7	17.1		0.529	9.7	1.5	I27	30.2	7.4		0.279	11.28	1.58
I04	4.6	19.2		0.104	2	1.5	I27	21.5	8.5		0.261	12.64	0.73
I06	25.5	8		0.287	11.28	33.38	I27	26.5	7.9		0.297	9.92	0.91
I06	21.5	7.6		0.267	12.8	42.48	I27	20.1	8.5		0.346	6.8	1.03
I06	28.3	8		0.238	12.4	33.38	I28	68.4	4.9		0.358	34.4	8.01
I06	21.3	7.5		0.283	12.24	37.02	I28	60.8	5.4		0.365	28.8	5.22
I06	25.5	7		0.287	12.72	35.2	I28	66	5.7		0.313	28.8	4.01
I06	21.6	7		0.317	14.8	33.98	I28	60	7.5		0.407	41.6	6.86
I07	27.1	5.5	76	0.347	20.24	2.31	I28	61.4	6.6		0.423	42.4	6.07
I07	23.1	7.1	77	0.414	13.68	1.64	I28	53.6	6.7		0.42	36.16	3.22
I07	26	6.8	77	0.409	12.88	2	I29	21.4	5.2		0.42	1.44	0.61
I07	23.3	7.1	76	0.353	10.48	2	I29	18.6	5.9		0.526	0.96	0.61
I07	30.7	7	77	0.454	10.72	2	I29	21.8	6.3		0.478	0.96	0.61
I07	25.7	7.3	77	0.503	13.92	2	I29	17.1	7.5		0.503	0.8	0.61
I08	62.4	7.5	81	0.272	71.6	2.91	I29	20.7	6.6		0.453	0.8	0.61
I08	44.3	8.8	79	0.285	52.32	1.21	I29	18.6	6.2		0.42	0.8	0.61
I08	43.3	9.3	75	0.238	56.4	2.1	I31	27.2	7.2		0.372	7.84	2.61
I08	42.1	9.7	74	0.226	52.64	2.1	I31	22.3	7.7		0.443	7.92	2.06
I08	53.3	9.9	77	0.264	47.76	2.1	I31	27.7	8.2		0.495	7.92	2.12
I08	38.9	10.1	77	0.299	43.04	2.1	I31	25.7	8.9		0.557	5.68	6.98
I09	87.9	7.7	76	0.355	84	2.31	I31	38	8.4		0.584	5.44	6.92
I09	66.1	9	73	0.279	66.64	1.09	I31	26.3	8.4		0.59	6.4	4.85
I09	57.7	9.5	73	0.256	65.84	1.7	I33	31.9	14.1		0.15	22	1.5
I09	59.1	9.8	72	0.266	71.6	1.7	I33	29.8	14.3		0.201	24.24	1.5
I09	84.1	9.6	74	0.271	76.88	1.7	I33	33.2	12.5		0.301	36.88	1.5
I09	69.2	9.9	74	0.235	66.48	1.7	I33	22.4	14.1		0.277	41.2	1.5
I10	38.5	10.4		0.545	18.72	1.52	I33	26.1	14.9		0.227	43.2	1.5
I10	40.6	10.8		0.535	17.84	1.09	I33	22.7	14.9		0.254	44.48	1.5
I10	35.3	11.1		0.506	12.08	1.09	I34	16.3	25.3		0.277	3.12	1.5
I10	37.4	10.8		0.486	10.56	1.4	I34	17	25.3		0.31	3.84	1.5
I10	31.8	9.6		0.428	14.64	1.03	I34	17.4	25.3		0.418	4.64	1.5
I10	33.8	9.7		0.424	12.48	0.61	I34	12.9	25.3		0.359	4.32	1.5
I11	37.5	3.3		0.339	17.12	2.18	I34	15.6	25.2		0.402	3.28	1.5
I11	33	5.1		0.395	17.12	2.49	I34	13.7	25.5		0.442	4.32	1.5
I11	41.2	6.4		0.394	16	2.55	I35	34.4	15.2		0.365	49.28	18.21
I11	28.3	6.8		0.42	14.72	2.43	I35	24.7	16.2		0.374	38.88	11.53
I11	31.4	6.7		0.439	13.28	2.41	I35	25.2	16.2		0.31	37.2	12.14
I11	28.6	6.8		0.464	12.24	2.41	I35	27.6	15.8		0.293	35.84	11.53
I12	30.9	3		0.297	16.24	2.55	I35	22.7	16.6		0.31	37.04	12.14
I12	21.4	4.9		0.325	13.04	1.52	I35	26.8	17.2		0.293	35.68	11.53
I12	34.6	5.4		0.388	15.2	1.09	I36	45.9	14.5		0.492	29.44	12.74
I12	19.9	5.3		0.348	11.2	1.72	I36	51.1	15.8		0.493	34.24	17.6
I12	26.2	5.9		0.434	8.48	1.72	I36	45	16.7		0.517	31.04	16.99
I12	20.8	6.4		0.491	9.04	1.72	I36	44.3	16.2		0.511	23.52	14.56
I13	16.7	0.3		0.378	4.72	1.94	I36	33.3	16.1		0.464	16.8	24.27
I13	11	2.2		0.345	4.24	1.5	I37	28	5		0.29	8.16	1.5
I13	15.7	3.4		0.313	4.08	1.5	I37	26.9	6.8		0.416	8.8	1.5
I13	9.7	4		0.347	2.8	1.5	I37	28.1	7.1		0.359	9.6	1.5
I13	12.5	4		0.357	2.48	1.5	I37	21.6	8		0.347	8	1.5
I13	11.3	4.1		0.386	1.52	1.5	I37	23.5	8.4		0.338	8.8	1.5
I14	40.7	12.3		0.473	42.24	15.17	I37	18.1	8.4		0.385	5.6	1.5
I14	34.5	13.1		0.546	37.04	15.17	I38	43	6.1		0.447	7.04	41.87
I14	44.2	13.5		0.52	31.04	18.21	I38	28.8	8		0.472	4	44.3
I14	35	13		0.511	32	18.81	I38	33.1	8.7		0.462	6.4	30.95
I15	83.5	14.6		0.423	120.8	125.01	I38	33.3	9.5		0.454	1.6	58.26
I15	68.1	16.2		0.488	77.04	155.96	I38	41.8	9.5		0.449	2.4	54.62
I15	70.7	16.1		0.427	61.04	158.38	I38	31.2	9.7		0.474	4	81.32
I15	66.4	15.6		0.349	64	154.14	I40	42.3	11.4		0.705	20.56	11.41
I15	72.6	15.6		0.503	35.84	138.36	I40	35.1	11.4		0.66	16.56	12.86
I16	19.6	6.5		0.493	14.4	4.85	I40	36	11.4		0.631	14.32	5.58
I16	15.5	6.5		0.474	9.04	3.64	I40	37.6	11.4		0.547	13.84	5.16
I16	19.6	7.1		0.542	8	4.25	I40	42.9	11.4		0.467	15.2	7.83
I16	12.3	6.7		0.47	6.48	3.03	I40	38	11.4		0.512	14.8	12.02
I18	82.1	13.6		0.352	32.5	83.74	I41	36.4	10.5		0.687	12.88	8.56
I18	70.2	14.2		0.37	32	149.89	I43	39.6	9		0.707	15.36	3.22
I18	70.5	15.4		0.45	31.44	101.95	I43	35.4	9		0.68	13.6	2.31
I19	118	9.7		0.691	8	95.27	I43	38.1	9		0.449	12.88	4.43
I19	95.8	9.7		0.664	24	112.26	I43	41.7	9		0.459	12.88	4.43
I19	83.5	9.7		0.728	25.6	107.41	I43	41.8	9		0.545	13.12	2.06
I20	37.6	13		0.494	42.88	1.5	I43	37.7	9		0.491	11.36	3.09
I20	39.7	13		0.38	42.88	1.5	I44	40.2	13.3		0.503	4.32	80.71
I20	48	13		0.218	42.4	1.5	I44	32.5	18.1		0.479	4.96	67.97
I21	101	9.6		0.471	171.68	8.92	I44	37.6	17.8		0.464	5.28	89.81
I21	95.1	11.9		0.527	147.68	8.5	I44	35.6	17.4		0.473	4.88	117.73
I21	126	12.8		0.567	133.6	14.93	I44	43.8	18.2		0.492	8.4	150.5
I23	44	16		0.654	5.84	36.41	I45	26.4	11.8		0.216	26.3	1.5
I23	40.9	15.9		0.639	6.48	47.94	I46	373	27.3		0.824	42.4	324.66
I23	45.2	15.5		0.644	6.64	41.87	I51	32.2	13.2		0.364	20	0.61
I23	39.7	15.8		0.643	6.48	37.62	I51	33.6	13.4		0.341	22.56	0.61
I23	48.2	16.1		0.644	6	39.44	I51	29.4	13.1		0.395	20.48	0.61
I23	42.1	16.2		0.683	5.68	40.05	I51	30.2	13.3		0.386	21.2	0.61
I24	38	14.1		0.18	11.28	2.61	I51	22.5	13.7		0.383	21.12	0.67
I24	28.6	14.1		0.221	11.6	2.49	I51	24.2	13.3		0.334	20.8	0.61
I24	48.8	13.9		0.275	11.28	2.85	I52	39	3.9		0.465	10.4	21.85
I24	32.1	14		0.262	11.36	3.34	I52	26.4	4.2		0.434	12.64	14.56
I24	55.8	14.2		0.258	12.24	3.16	I52	22.4	5.8		0.405	27.44	11.23
I24	33.8	14.6		0.291	12.4	3.09	I52	23.9	5.9		0.396	32.88	6.68
I25	118	22.8		0.538	8.88	80.1	I52	17.4	6.8		0.483	20.32	5.28
I25	138	23.9		0.525	6.64	60.08	I52	26.3	6.2		0.503	22.32	7.34

**Table 6:** MICAT data considered in the study.

Code	1st Year Corrosion, μm	T, °C	RH, %	TOW, Annual Fraction	Precipitation, mm/y	Deposition Rates, mg/m^2^.d	
						SO_2_	Cl
M01	54.8	13.9	79	0.708	805	2	40.2
M01	66	14.5	80	0.736	1226	2	70
M02	14.73	16.9	74	0.538	1377	9	1.5
M03	5.7	21.2	75	0.643	2167	2	1.50
M04	4.9	18.8	50	0.103	80	2	1.50
M06	25.3	17	78	0.593	1178	6.2	1.5
M06	28.8	16.7	77	0.565	1263	8.2	1.5
M06	30.1	16.6	78	0.631	1361	6.2	1.5
M07	8.6	21.5	74	0.482	847	0.8	8.9
M07	11.5	20.9	75	0.482	1167	1.3	7.4
M07	13.1	21.2	75	0.482	996	1.7	1.60
M08	52.5	23.8	89	0.482	1122	23.8	8.6
M08	47.3	22.9	91	0.482	1471	20.7	6.8
M08	48.5	23	90	0.482	1444	24.5	5.2
M09	159.8	24.8	77	0.582	605	9.5	359.8
M09	194.7	24.5	79	0.582	985	5.3	174.8
M09	141.7	24.2	77	0.582	716	4.4	167.70
M10	98.7	22.7	73	0.579	960	40.4	4.50
M10	161.2	22.9	71	0.579	870	57.4	9.20
M10	216.9	22.6	79	0.579	1133	65.8	10.80
M11	301.9	22.1	80	0.579	1689	2.6	113.20
M13	127.1	20.1	80	0.598	1353	55.85	20.21
M13	61.2	23.1	78	0.598	1369	44.09	14.22
M13	73.1	21	82	0.598	1305	30.51	14.67
M14	19.4	26.1	88	0.682	2395	2	1.50
M16	12.9	20.4	69	0.442	1440	2	1.50
M17	17.3	25.9	77	0.172	1392	2	1.50
M18	4.9	26.6	90	-	2096	2	1.50
M19	16	27.6	85	0.989	940	7.8	43.60
M19	30.6	27.6	87	0.966	940	14.2	69.00
M19	54	28.2	87	0.975	940	8.9	69.50
M20	17	11.5	90	1	1800	0.6	1.50
M21	19.6	27	76	0.33	900	0.3	1.50
M22	61.6	27.6	80	0.562	1598	6.3	38.7
M23	371.5	25.3	88	0.763	3531	3.5	376
M24	69.3	22.9	88	0.838	3677	4	20.60
M25	16.6	18.9	83	0.695	1780	2.4	12.10
M26	36.1	25.2	80	0.571	1591	37.1	15.8
M26	26.4	25.4	79	0.571	1303	36.5	10.9
M26	29	24.7	79	0.571	1321	19.8	10.9
M26	32.3	25.5	79	0.571	1129	25.6	14.3
M26	31.3	25.4	79	0.571	1305	41	10.10
M26	29.2	24.7	79	0.571	1540	24.7	8.20
M26	27.3	25.2	79	0.571	1415	18.5	18.10
M26	32.8	25.1	79	0.571	1064	49.2	7.40
M27	391.1	25.2	80	0.571	1591	24.5	99.10
M27	213.7	25.4	79	0.571	1303	13.5	115.60
M27	173.6	24.7	79	0.571	1321	25.1	123.30
M27	171.9	25.5	79	0.571	1129	20.4	96.00
M27	391.2	25	79	0.571	1108	32.8	50.40
M27	126.5	25.4	79	0.571	1305	18.9	111.40
M27	71.6	25.7	79	0.571	1540	19.9	108.80
M27	84.8	24.2	79	0.571	1415	17.7	97.70
M27	188.9	25.1	80	0.571	1064	40.4	81.80

**Table 7:** Characteristics of the corrosion and environmental data used in the statistical treatment.

Type of Atmosphere	Variable	Smallest Value	Largest Value
Non marine	C (µm)	0.51	87.9
	T (°C)	3.1	27
	RH (%)	35	90
	SO_2_ (mg/m^2^.d)	0.08	84
Marine	C (µm)	8.6	411.2
	T (°C)	3.9	28.2
	TOW (annual fraction)	0.16	0.99
	SO_2_ (mg/m^2^.d)	0.3	171.7
	Cl (mg/m^2^.d)	3.03	376

**Table 8:** Published dose/response (D/R) functions for first-year corrosion of carbon steel.

Type of Atmosphere	Programme	Ref.	D/R Function	N	R^2^
Non marine	ICP/UNECE (for weathering steels)	[18]	C = 34[SO_2_]^0.13^ exp{0.020 RH + f(T)} where f(T) = 0.059(T − 10) when T ≤ 10 °C, otherwise f(T) = −0.036 (T − 10)	148	0.68
All atmospheres (marine and non-marine atmospheres)	ISOCORRAG	[35]	C = 0.091 [SO_2_]^0.56^ TOW^0.52^ exp(f(T)) + 0.158[Cl]^0.58^ TOW^0.25^ exp(0.050 T) where f(T) = 0.103 (T − 10) when T ≤ 10 °C, otherwise f(T) = −0.059 (T − 10)	125	0.85
			C = 1.77 [SO_2_]^0.52^ exp(0.020 RH) exp(f(T)) +0.102[Cl]^0.62^ exp(0.033 RH +0.040 T) where f(T) = 0.150 (T − 10) when T ≤ 10 °C, otherwise f(T) = −0.054 (T − 10)	128	0.85
	MICAT	[7]	C = −0.44 + 6.38 TOW + 1.58[SO_2_] + 0.96[Cl]	172	0.56
	ISOCORRAG/MICAT Including data from Russian sites in frigid regions	[1]	C = 0.085 × SO_2_^0.56^ × TOW^0.53^ × exp(f) + 0.24 × Cl^0.47^ × TOW^0.25^ × exp(0.049 T) f(T) = 0.098 (T − 10) when T ≤ 10 °C, otherwise f(T) = −0.087 (T − 10)	119	0.87

N = number of data.

**Table 9:** D/R functions for first-year corrosion of carbon steel. Contribution of the information supplied by each of the international programmes to the overall D/R functions (Equations (3) and (7)) using the integrated database.

Type of Atmosphere	Programme	N	Significant Variables	D/R Function	R^2^
Non-marine atmospheres	Overall D/R function (Equation (3)) (ICP/UNECE + ISOCORRAG + MICAT)	333	SO_2_, RH, T	C= 0.82 SO_2_ + 0.26 RH + 0.15 T	0.725
	ICP/UNECE	269	SO_2_, RH, T	C= 0.74 SO_2_ + 0.37 RH + 0.21 T	0.674
	ISOCORRAG	18	SO_2_	C= 0.93 SO_2_	0.867
	MICAT	46	SO_2_, T	C= 0.49 SO_2_ + 0.46 T	0.398
Marine atmospheres	Overall D/R function (Equation (7)) (ISOCORRAG + MICAT)	206	Cl, SO_2_, TOW	C= 0.62 Cl + 0.22 SO_2_ + 0.15 TOW	0.474
	ISOCORRAG	97	Cl, SO_2_, TOW	C= 0.57 Cl + 0.31 SO_2_ + 0.31 TOW	0.582
	MICAT	109	Cl, SO_2_,	C= 0.69 Cl + 0.30 SO_2_	0.538

N = number of data.
